# Resonant Photoionization of CO_2_ up to the Fourth Ionization Threshold

**DOI:** 10.1021/acs.jpca.3c06947

**Published:** 2023-12-20

**Authors:** Prateek Pranjal, Jesús González-Vázquez, Roger Y. Bello, Fernando Martín

**Affiliations:** † Instituto Madrileño de Estudios Avanzados en Nanociencia (IMDEA-Nanociencia), Cantoblanco, 28049 Madrid, Spain; ‡ Departamento de Química, Módulo 13, Facultad de Ciencias, Universidad Autónoma de Madrid, 28049 Madrid, Spain; § Departamento de Química Física Aplicada, Universidad Autónoma de Madrid, 28049 Madrid, Spain

## Abstract

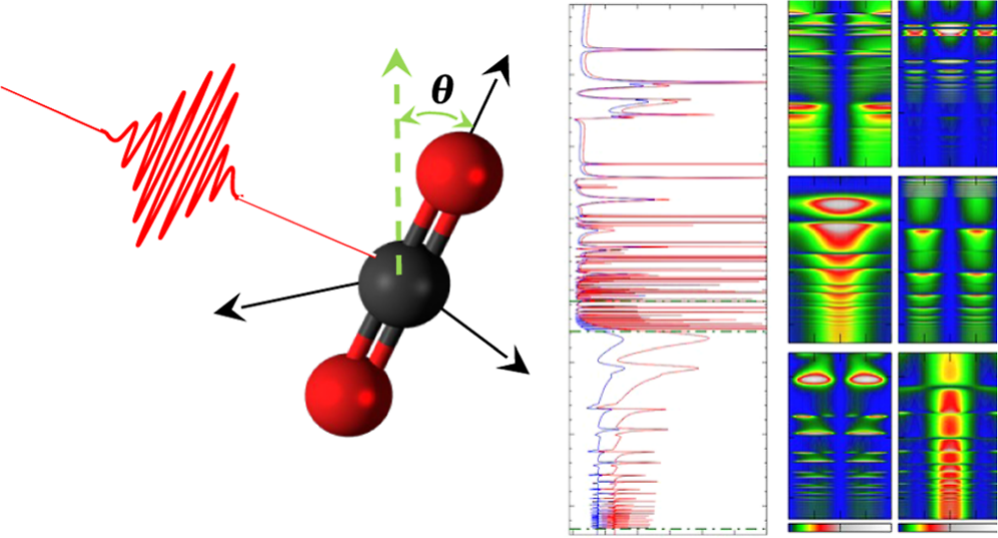

We present a comprehensive theoretical study of valence-shell photoionization of the CO_2_ molecule by using the XCHEM methodology. This method makes use of a fully correlated molecular electronic continuum at a level comparable to that provided by state-of-the-art quantum chemistry packages in bound-state calculations. The calculated total and angularly resolved photoionization cross sections are presented and discussed, with particular emphasis on the series of autoionizing resonances that appear between the first and the fourth ionization thresholds. Ten series of Rydberg autoionizing states are identified, including some not previously reported in the literature, and their energy positions and widths are provided. This is relevant in the context of ongoing experimental and theoretical efforts aimed at observing in real-time (attosecond time scale) the autoionization dynamics in molecules.

## Introduction

The development of attosecond VUV/XUV pulses has opened new doorways for imaging and steering electron dynamics in many-electron systems, in particular, molecular systems.^1−12^ Recent examples also include retrieving real-space movies of the internal motion in molecules,^13,14^ monitoring the birth of a photoelectron in helium,^15^ extracting photoionization time delays of molecules in the vicinity of shape and Feshbach resonances,^8,10,16−19^ and the observation of correlation-driven charge migration in a DNA building block.^11^ The use of these light sources usually leads to ionization, where in addition to the photoelectron ejection, other processes involving two or more electrons can also take place, e.g., autoionization of Feshbach resonances, ionization leaving the remaining ion in an excited state (shakeup), inner-shell ionization followed by Auger decay, and Auger decay combined with shakeup. Thus, any comprehensive theoretical description of these types of experiments requires a fully correlated treatment of the electronic continuum. In addition, photoionization processes of many-electron systems are also sensitive to interchannel couplings, including those between energetically open and closed channels.

All of the above has prompted the development of advanced theoretical methods based on different approximations to account for electron correlation in the molecular continuum. Among these methods, the multichannel Schwinger configuration interaction method (MCCI),^20−22^ the variational Complex-Khon^23,24^ method, the UK Molecular R-matrix,^25^ and the XCHEM method^26,27^ are well established nowadays. In particular, the XCHEM approach combines standard quantum chemistry techniques with a single-center hybrid Gaussian-B-spline basis (GABS),^26^ providing a fully correlated description of the electronic continuum at a level similar to that provided by quantum chemistry packages in bound state calculations. This makes XCHEM particularly well suited to study photoionization processes in many-electron systems. In previous works, XCHEM has been shown to provide accurate photoionization spectra in the resonance regions of He, Ne, and Ar atoms,^27−29^ and small molecules such as N_2_, O_2_, and more recently H_2_O.^30−33^

**Figure 1:**
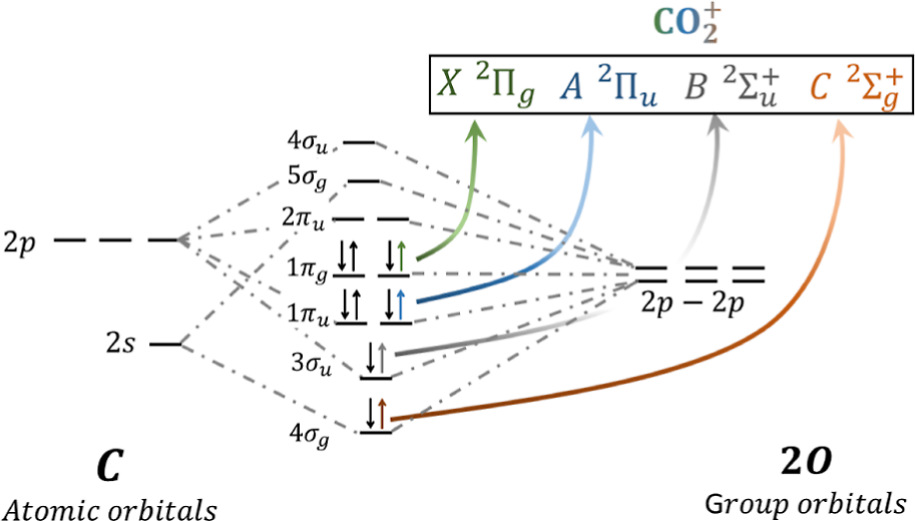
Schematic representation of the molecular orbitals of CO_2_ and the cationic electronic states resulting from the removal of an electron from different molecular orbitals.

In the present paper, we take advantage of the XCHEM capabilities to study valence-shell one-photon single ionization of the CO_2_ molecule, for photon energies between the first and fourth ionization thresholds. While the energy region above the fourth ionization threshold has been extensively studied, both theoretically and experimentally,^34−42^ the existing theoretical information for energies below this threshold is rather scarce. This is because, despite its apparent simplicity, the CO_2_ molecule presents very rich and complex photoionization dynamics. As schematically depicted in Figure 1, the removal of an electron from the 1π_g_ (HOMO), 1π_u_ (HOMO – 1), 3σ_u_ (HOMO – 2), and 4σ_g_ (HOMO – 3) molecular orbitals leaves the remaining CO_2_^+^ cation in the X^2^Π_g_, A^2^Π_u_, B^2^Σ_u_^+^, or C^2^Σ_g_^+^ electronic states lying at 13.778, 17.314, 18.077, and 19.394 eV, respectively.^43^ The fourth excited state of the CO_2_^+^(C^2^Σ_g_^+^) cation lies just 6 eV above the energy of the CO_2_^+^ (X^2^Π_g_) ground state. Consequently, several series of Rydberg autoionizing states converging to the different ionization thresholds are expected to overlap in this energy region. Total single-photon ionization cross sections in this energy region have been previously measured in photoabsorption experiments using synchrotron radiation sources.^44−50^ In these experiments, several series have been identified, namely, the so-called Tanaka–Ogawa, Lindholm, Henning sharp and diffuse, “absorption”, “apparent emission”, and “weak absorption” series. However, their assignment and characterization remain uncertain. In addition, most of these resonances have not been theoretically described so far.

In this work, we have evaluated one-photon single-ionization fully differential cross sections and analyzed the effect of the autoionizing Rydberg states lying below the fourth ionization threshold. We have identified several series of Rydberg states, including some not observed in the existing experiments, e.g., the 1π_u_^–1^*nd*π_g_ and 1π_u_^–1^*ns*σ_g_ series converging to the second ionization threshold, as well as the 3σ_u_^–1^*nd*π_g_ and 4σ_g_^–1^*nf*π_u_ series converging to the third and fourth ionization thresholds, respectively. The members of the different Rydberg series have been characterized and, in most cases, their corresponding energy positions and widths are given. Finally, the fully differential cross sections for photon energies above the fourth ionization thresholds are provided and compared with experimental and theoretical results found in the literature. The good agreement found at these higher energies gives additional support to our predictions at lower energies.

## Methods and Computational Details

One-photon ionization cross sections, molecular-frame photoelectron angular distributions (MFPADs), and β asymmetry parameters were calculated at a fixed internuclear distance of *R* = 2.1943 au using the XCHEM methodology.^27^ This methodology has been explained in detail elsewhere,^26,27,30−32^ so only the computational details will be given here.

The initial set of orbitals used in the XCHEM calculations are the CO_2_^+^ (X^2^Π_g_) ground-state natural orbitals. The cationic ground state was obtained from a complete active space configuration interaction (CAS-CI) calculation, where the active space included the first five σ_g_, three σ_u_, two π_u_, and one π_g_ orbitals with the 1–2σ_g_ and 1σ_u_ core orbitals always doubly occupied (see Figure 1). These orbitals were optimized using a restricted active space SCF (SA-RASSCF) calculation using MOLCAS^51^ where they form the active space and in which only the CO_2_^+^ (X^2^Π_g_) ground state was included in the state average. The one-electron basis was aug-cc-pVTZ.^52^ The neutral ground state was computed by constructing the (*N* + 1)-electron configuration state functions (CSFs) using the same orbitals as in the MOLCAS calculations of the cation target state. In the close-coupling calculation, the four lowest channels, X^2^Π_g_, A^2^Π_u_, B^2^Σ_u_^+^, and C^2^Σ_g_^+^, were included. We note that a similar active space was used in a previous work,^42^ obtaining ionization potentials in very good agreement with the experimental values (see Table 1).

**Table 1:** Target Energies Absolute and Relative to the CO_2_^+^ Ground State^a^

state	XCHEM (eV)	UKRMol (eV)	experiment (eV)
X^2^Π_g_	0.00 (13.51)	0.00 (14.85)	0.0 (13.80)
A^2^Π_u_	4.08 (17.59)	3.97 (18.82)	3.8 (17.60)
B^2^Σ_u_^+^	4.29 (17.80)	4.45 (19.27)	4.3 (18.10)
C^2^Σ_g_^+^	5.66 (19.17)	5.77 (20.59)	5.6 (19.40)

a The present results are compared to the UKRMol results, obtained using the same active space^42,53^ and the experimental values.^54^

The set of monocentric GABS basis functions^26^ used to describe the photoelectron is placed at the system origin, with the B-splines being nonzero for radii *r* > *R*_0_ and the monocentric Gaussian being nonzero for a radii *r* < *R*_1_ such that *R*_0_ ≤ *R*_1_. The B-splines part of the basis consists of a set of 800 B-splines of order *k* = 7 extending from *R*_0_ = 8 au up to *R*_max_ = 400 a_0_ with  ≤ 7. The Gaussian part contains a set of 22 even tempered functions , with α_*i*_ = α_0_β^*i*^ (α_0_ = 0.01, β = 1.46, *i* = 0, 1, ..., 21), and ζ = 0,  ≤ 7.

In this work, our focus has been on the energy region between the first (X^2^Π_g_) and fourth (C^2^Σ_g_^+^) ionization thresholds. Since only four channels have been included in the close-coupling calculation, no autoionizing resonances are expected to be present beyond the fourth (C^2^Σ_g_^+^) ionization threshold. No energy shift has been applied to the data, hence, the positions of the resonance peaks are slightly shifted to lower photon energies when compared to experimental results.^43^

### Resonance Analysis

The energy positions and widths of the Rydberg autoionizing states have been calculated from the photoionization spectra. The total phase of the scattering states was fitted to the analytical expression^55^ in eq 1, which describes the behavior of the scattering phase in the vicinity of an autoionizing state
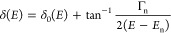
1where δ_0_ is a smoothly varying background, and *E*_n_ and Γ_n_ are the resonance position and width, respectively. The resonances have to be isolated to be able to employ this equation. Thus, in case of partially overlapping resonances, only an estimate of the resonance position and width can be given.

The autoionizing states have been assigned to different Rydberg series considering the available literature and using the Rydberg equation
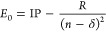
2where *E*_0_ is the resonance position, IP is the ionization potential of the state, *R* is the Rydberg constant, *n* is the principal quantum number of the Rydberg electron, and δ is the quantum defect. The quantum defect δ correlates with the orbital angular momentum *l*. Larger values (δ ∼ 1) are expected for *l* = 0, while small values (δ ∼ 0) are expected for *l* = 2, 3.

## Results and Discussions

### Photoionization at Low Photoelectron Energies

**Figure 2:**
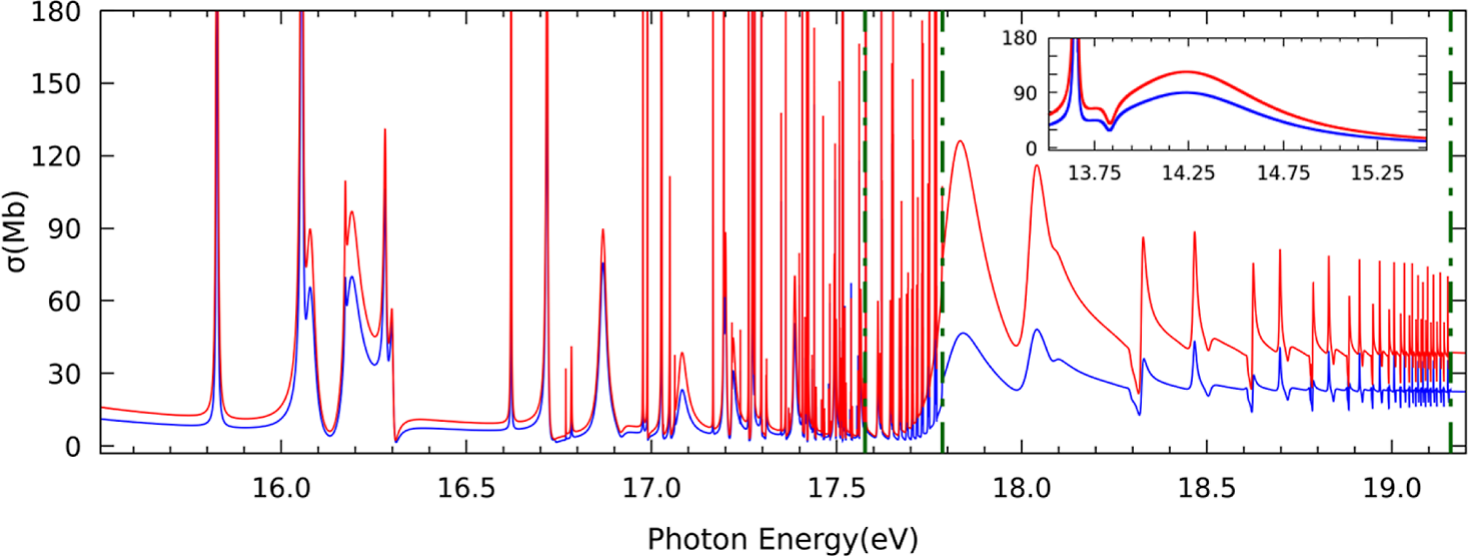
Computed total photoionization cross sections of CO_2_ as a function of the photon energy. The comparison between length (red solid line) and velocity (blue solid line) gauges is also depicted. Green dashed line: energy position of each ionization threshold.

Figure 2 depicts the total photoionization cross section for one-photon absorption for photon energies between the first and fourth ionization thresholds. The cross sections calculated in length and velocity gauges are generally in good agreement, reflecting the quality of the used basis set. As observed, the cross section is characterized by several autoionizing states. As no shift in energy has been applied to the data, the positions of the autoionizing Rydberg states might appear slightly shifted in photon energy when compared to experimental results. In the following, further consideration is dedicated to the characterization and assignment of these autoionizing Rydberg states.

**Figure 3:**
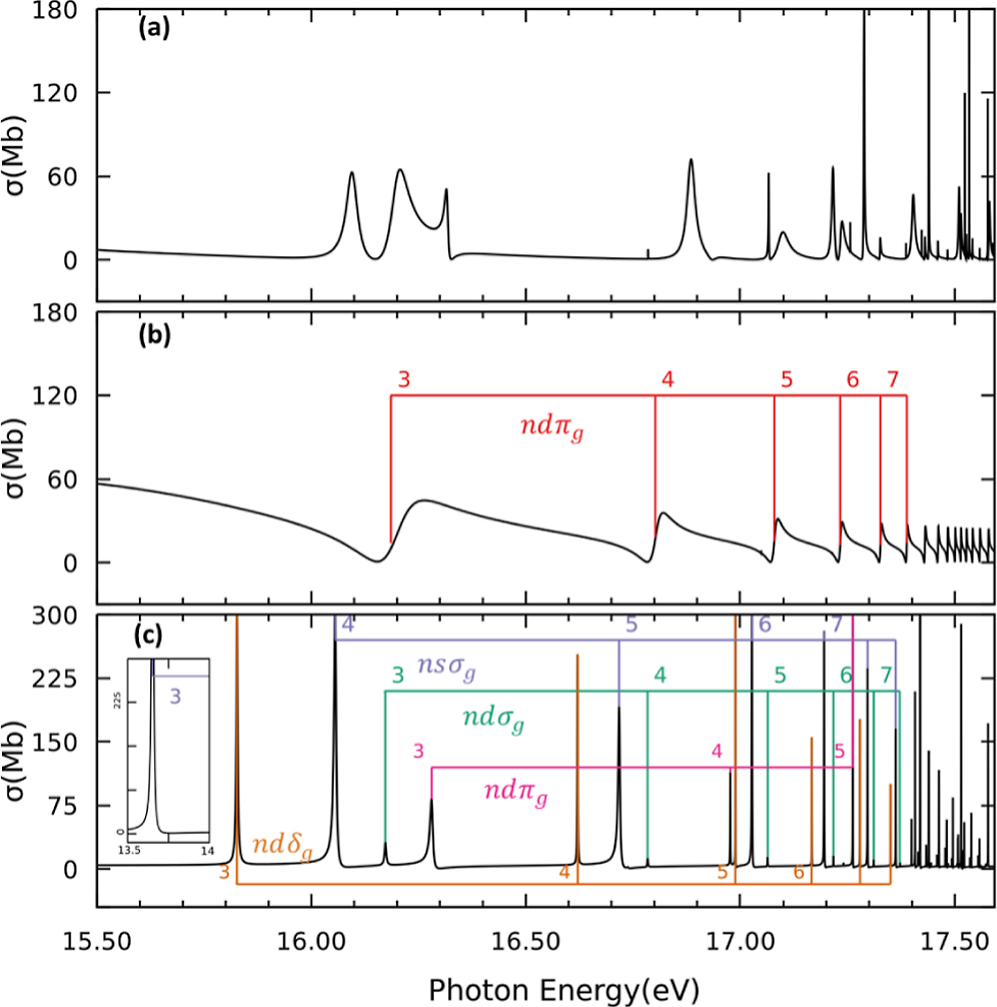
Partial photoionization cross sections for photon energies between the first (X^2^Π_g_) and second (A^2^Π_u_) ionization thresholds. (a) Molecular axis is placed parallel to the light polarization vector, i.e., ^1^Σ_u_^+^ final symmetry. (b) Model calculation limiting the number of channels included in the close coupling to the first two ionization thresholds for the ^1^Σ_u_^+^ final symmetry. (c) Molecular axis is placed perpendicular to the light polarization vector, i.e., ^1^Π_u_ final symmetry. The corresponding resonances are indicated with nl labels.

**Figure 4:**
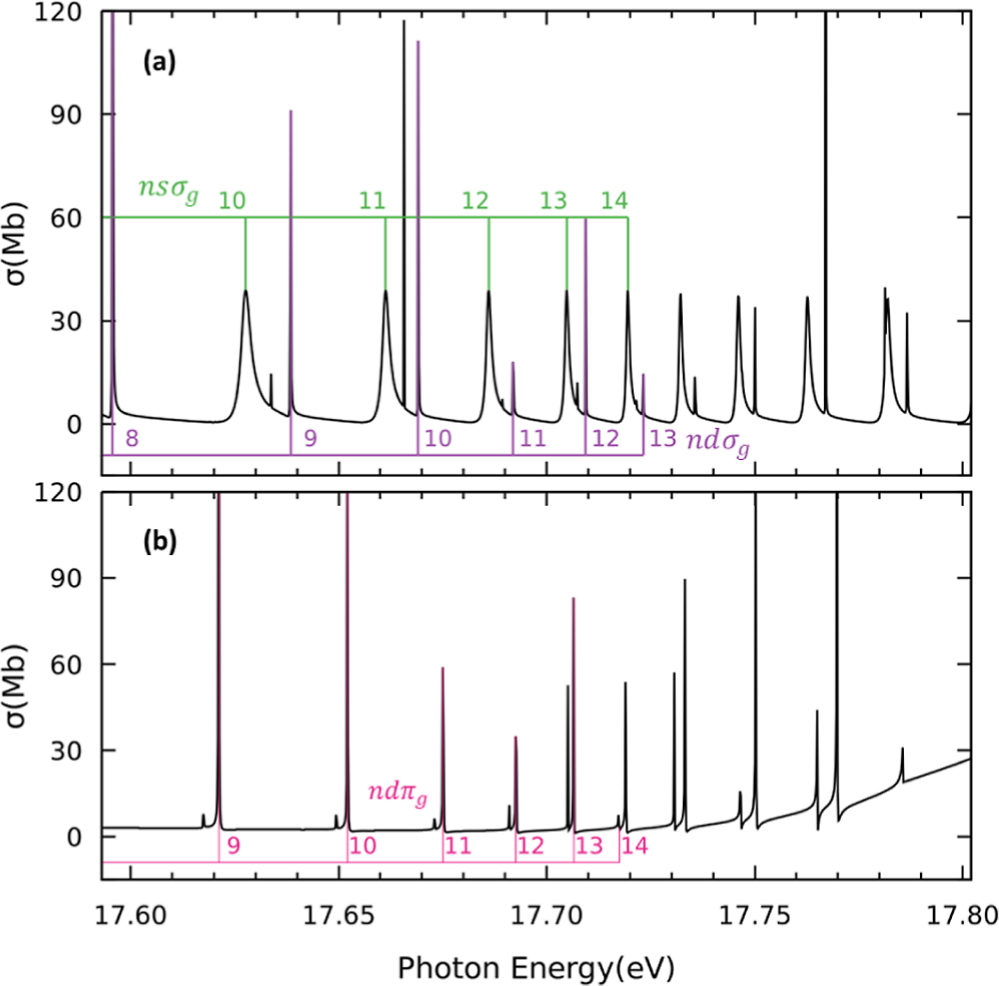
Same as Figure 3, but for photon energies between the second (A^2^Π_u_) and third (B^2^Σ_u_^+^) ionization thresholds. (a) Molecular axis is placed parallel to the light polarization vector. (b) Molecular axis is placed perpendicular to the light polarization vector.

Figures 3a and 4a present the partial photoionization cross section from the ground state leading to states of ^1^Σ_u_^+^ symmetry between the first and second, and between second and third ionization thresholds, respectively. The cross section between the second and third ionization thresholds (Figure 4a) is characterized by several autoionizing states associated with the Henning sharp 3σ_u_^–1^*nd*σ_g_ and diffuse 3σ_u_^–1^*ns*σ_g_ series converging to the third ionization threshold B^2^Σ_u_^+^.^44−49^ All Rydberg series identified are summarized in Tables 2–4, where the estimated energy positions and autoionization widths using eq 2 along with the value of δ characterizing the quantum defect are also presented. As observed, a quantum defect around δ = −0.127 and δ = 1.167 have been obtained for the Henning sharp and diffuse series, respectively. These values are in good agreement with those reported in refs (48) and (49), thus confirming the assignment. The spectrum between the first and second ionization thresholds (Figure 3a) appears to be dominated by three different series of autoionizing Rydberg states. Based on the analysis of Figure 4a, we see that some resonances correlate to low energy members of the Henning sharp and diffuse series. In order to further characterize this energy region, we performed additional calculations limiting the number of channels included in the close coupling to the first two ionization thresholds. Thus, in this scenario, resonances converging to higher ionization thresholds are not expected to appear in the spectrum. The result of these calculations is presented in Figure 3b. As observed, the cross section indeed features just a single Rydberg series, identified as 1π_u_^–1^*nd*π_g_ series converging to the second ionization threshold A^2^Π_u_. The lower members of the series exhibit broad Fano profiles with widths up to ∼100 meV (see Table 2). Therefore, the members of the 1π_u_^–1^*nd*π_g_ and the Henning sharp and diffuse series overlap in energy, making the full characterization of Rydberg states in this energy region unfeasible.

**Table 2:** *nd*π_g_, *nd*σ_g_, *nd*δ_g_, and *ns*σ_g_ Rydberg Series Converging to the Second CO_2_^+^ (A^2^Π_u_) Ionic State^a^

*n*	calculated *E* (eV)	estimated E. (eV)	width Γ (meV)
	*nd*π_g_^1^Σ_u_^+^	δ = 0.159	
3	16.1860	16.2296	100.49
4	16.8015	16.8065	31.41
5	17.0791	17.0819	15.15
6	17.2328	17.2344	8.46
7	17.3267	17.3276	5.23
	*nd*(σ_g_*or* δ_g_)^1^Π_u_	δ =–0.151	
3	16.1729	16.2065	5.55
4	16.7836	16.7871	1.85
5	17.0630	17.0640	
6	17.2171	17.2171	
7	17.3109	17.3107	
	*nd*(σ_g_*or* δ_g_)^1^Π_u_	δ = 0.241	
3	15.8260	15.7889	3.86
4	16.6203	16.6137	1.31
5	16.9807	16.9759	
6	17.1664	17.1665	
7	17.2787	17.2789	
	*ns*σ_g_^1^Π_u_	δ = 1.031	
3	13.6529	14.0668	11.60
4	16.0534	16.0331	6.09
5	16.7142	16.7130	5.27
6	17.0269	17.0257	1.04
7	17.1947	17.1948	

a The final symmetry is specified in each case. The corresponding resonances are indicated with nl labels in Figure 3. The estimated energies were obtained using eq 2 and the value of the quantum defect δ. The calculated energy positions and widths were obtained by fitting the corresponding scattering phase to eq 1.

**Table 3:** Same as Table 2, for *ns*σ_g_, *nd*σ_g_, and *nd*π_g_ Rydberg Series Converging to the Third CO_2_^+^ (B^2^Σ_u_^+^) Ionic State^a^

*n*	calculated *E* (eV)	estimated *E* (eV)	width Γ (meV)
	*ns*σ_g_^1^Σ_u_^+^	δ = 1.167	
4		16.1065	
5		16.8759	
6		17.2195	
7		17.4021	
8		17.5106	
9		17.5803	
10	17.6274	17.6277	2.81
11	17.6611	17.6613	2.03
12	17.6859	17.6861	1.52
	*nd*σ_g_^1^Σ_u_^+^	δ =–0.127	
3		16.4108	
4		17.0033	
5		17.2845	
6		17.4396	
7		17.5342	
8	17.5979	17.5961	
9	17.6415	17.6387	
10	17.6681	17.6694	
	*nd*π_g_^1^Π_u_	δ =–0.094	
3	16.2663	16.3648	8.34
4	13.6931	16.9741	
5	17.2624	17.2615	
6	17.4194	17.4194	
7	17.5164	17.5154	

a The corresponding resonances are indicated with nl labels in Figures 3 and 4.

**Table 4:** Same as Table 2, for *np*σ_u_, *nf*σ_u_, *np*π_u_, and *nf*π_u_ Rydberg Series Converging to the Fourth CO_2_^+^ (C^2^Σ_g_^+^) Ionic State^a^

*n*	calculated E. (eV)	estimated E. (eV)	width Γ (meV)
	*np*σ_u_^1^Σ_u_^+^	δ = 0.443	
4	18.1021	18.0994	34.73
5	18.4679	18.5199	18.10
6	18.7361	18.7345	9.84
7	18.8597	18.8587	5.98
8	18.9377	18.9370	3.88
	*nf*σ_u_^1^Σ_u_^+^	δ = 0.024	
4	18.3099	18.3146	12.15
5	18.6261	18.6257	6.49
6	18.7970	18.7943	4.51
7	18.8955	18.8957	3.12
8	18.9612	18.9614	2.04
	*np*π_u_^1^Π_u_	δ = 0.568	
4	17.9971	18.0040	13.56
5	18.4486	18.4664	10.31
6	18.6973	18.6979	5.00
7	18.8298	18.8301	2.80
8	18.9124	18.9126	1.74
	*nf*π_u_^1^Π_u_	δ =–0.038	
4	18.3126	18.3244	1.08
5	18.6174	18.6228	
6	18.7827	18.7857	
7	18.8823	18.8842	
8	18.9471	18.9483	

a The corresponding resonances are indicated with nl labels in Figure 5.

Figure 3c shows the partial photoionization cross section from the ground state leading to states of ^1^Π_u_ symmetry between the first and second ionization thresholds. The cross section features four different Rydberg series. Three of them, identified as 1π_u_^–1^*ns*σ_g_, 1π_u_^–1^*nd*δ_g_, and 1π_u_^–1^*nd*σ_g_, are found to converge to the second ionization threshold A^2^Π_u_. In particular, the 1π_u_^–1^*ns*σ_g_ series characterized by a quantum defect of δ = 1.031, is assigned to the Tanaka–Ogawa series.^45−49^ The assignment is made based on the value of quantum defect and the fact that the *n* = 3 member appears at 13.65 eV, very close to the ionization threshold (see insets in Figures 2 and 3c). As observed, the 1π_u_^–1^*nd*σ_g_ series presents very low cross sections compared to those of the 1π_u_^–1^*nd*δ_g_ and 1π_u_^–1^*ns*σ_g_, and should not be visible experimentally. Taking this into account, we tentatively assign the 1π_u_^–1^*nd*δ_g_ series to the Lindholm series.^45−49^ The 3σ_u_^–1^*nd*π_g_ series converging to the third ionization threshold B^2^Σ_u_^+^, has not been observed experimentally. Higher energy members of this series can be observed in Figure 4b, which depicts the partial cross section from the ground state leading to states of ^1^Π_u_ symmetry between the second and third ionization thresholds.

Figure 5a,b shows the partial photoionization cross section between the third and fourth ionization thresholds from the ground state leading to states of ^1^Σ_u_^+^ and ^1^Π_u_ symmetries, respectively. While the cross section exhibits four different Rydberg series converging to the fourth ionization threshold C^2^Σ_g_^+^, just three have been observed experimentally in this energy region.^45,48,49^ Two of them, the “absorption” and “apparent emission” series are identified as the 4σ_g_^–1^*np*π_u_ and 4σ_g_^–1^*np*σ_u_ series, respectively. In contrast, the “weak absorption” series assignment is still under debate. The members of the 4σ_g_^–1^*nf*σ_u_ series are generally broader than those of the 4σ_g_^–1^*nf*π_u_, and thus, more prone to be observed in experimental photoionization spectra. Based on this analysis, we tentatively assign the 4σ_g_^–1^*nf*σ_u_ series as the “weak absorption” series. There is an additional broad peak, labeled as “X” in Figure 5b, lying just at the ionization threshold (see Figure 2), making its assignment and further characterization unfeasible.

**Figure 5:**
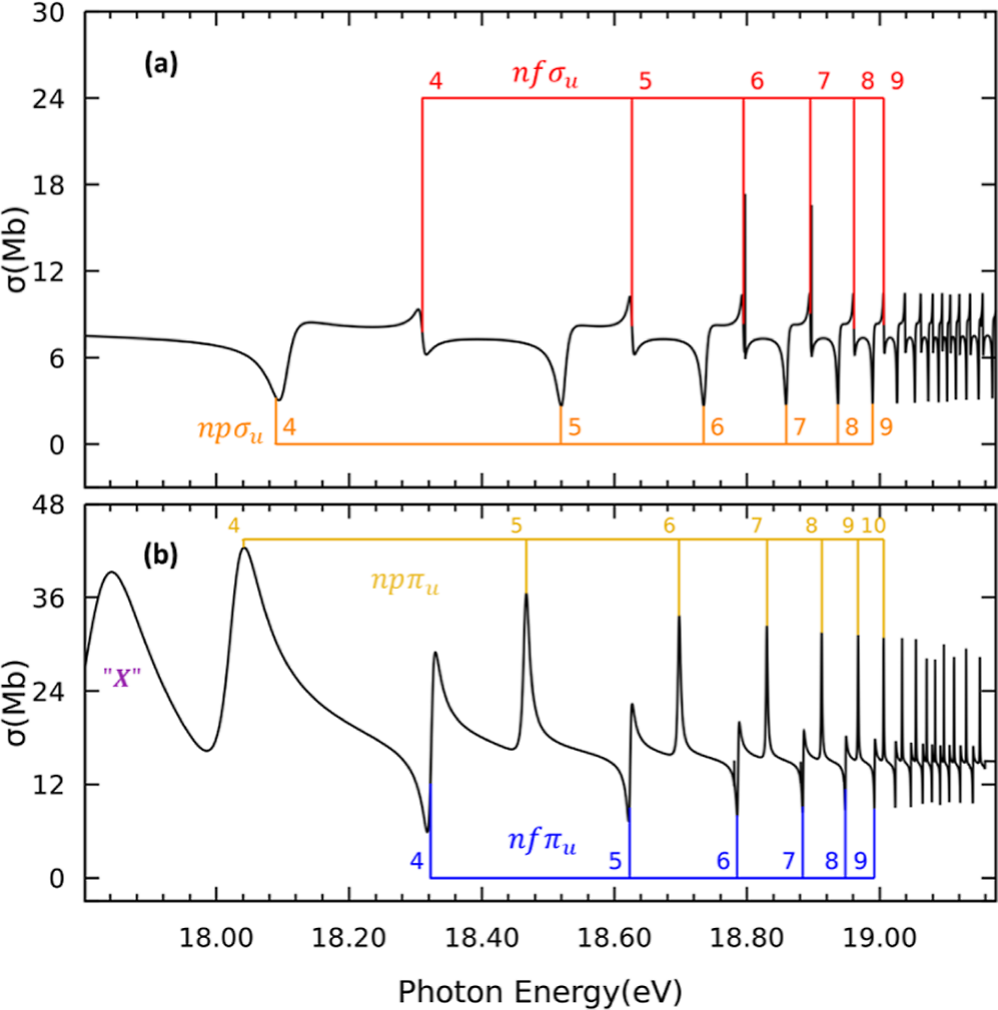
Same as Figure 3, but for photon energies between the third (B^2^Σ_u_^+^) and fourth (C^2^Σ_u_^+^) ionization thresholds. (a) Molecular axis is placed parallel to the light polarization vector. (b) Molecular axis is placed perpendicular to the light polarization vector.

We note that previous photoabsorption experiments in CO_2_^44,45,56^ have pointed out the existence of rather long vibrational progressions associated with the different series of Rydberg states. However, such vibrational progressions are difficult to resolve in the corresponding photoionization spectra.^43,47,50,56^ This is probably due to (i) the limited energy resolution in photoionization experiments compared to photoabsorption experiments, (ii) the overlap between different vibrational progressions, and (iii) the fact that most of these resonances are long-lived so that their signature is ultimately washed out by nuclear motion. In contrast, our fixed-nuclei calculations allow for a more straightforward assignment of the resonance series. In addition, comparison with photoionization experiments with low energy resolution should be more straightforward.

**Figure 6:**
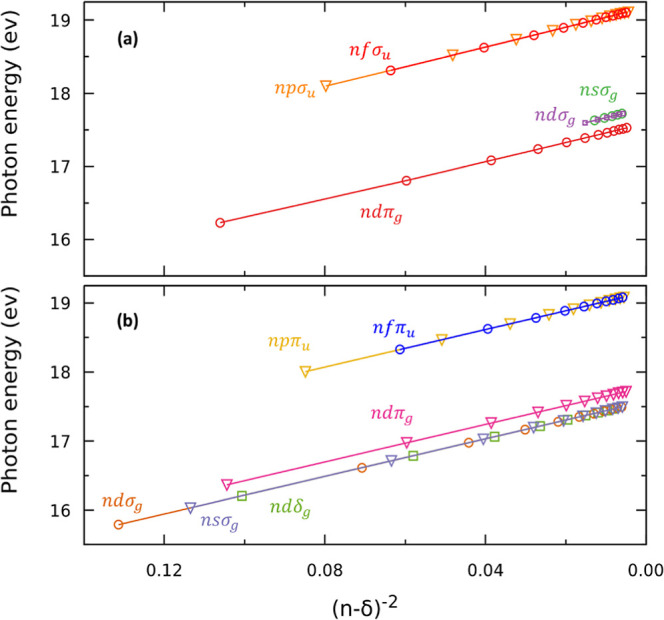
Energies of the different Rydberg series identified in the computed photoionization cross section as a function of (*n* – δ)^−2^. (a) Light polarization vector parallel to the molecular axis, i.e., ^1^Σ_u_^+^ final symmetry. (b) Light polarization vector perpendicular to the molecular axis, i.e., ^1^Π_u_ final symmetry.

Figure 6a,b presents the calculated energy positions for the different Rydberg series as a function of the effective quantum number n* = *n* – δ for the ^1^Σ_u_^+^ and ^1^Π_u_ final symmetries, respectively. A nearly perfect (n*)^−2^ scaling is observed in agreement with eq 2, thus confirming the validity of the assignment. For the higher n*, the density of resonances increases significantly, so the assignment could be misleading. As expected, the autoionization widths decrease with the effective quantum number n*. Some of the resonances observed in the photoionization cross sections are particularly narrow, with widths Γ < 10 meV, especially just below the different ionization thresholds. Therefore, in Tables 2–4 and in Figure 6a,b, only Rydberg states with widths Γ ≥ 1 meV are shown. Such sharp resonances are usually not observed experimentally due to both the spectral resolution and the effect of the nuclear degrees of freedom (not considered in these calculations). However, most of the series identified here have been previously observed, although not characterized, experimentally.

**Figure 7:**
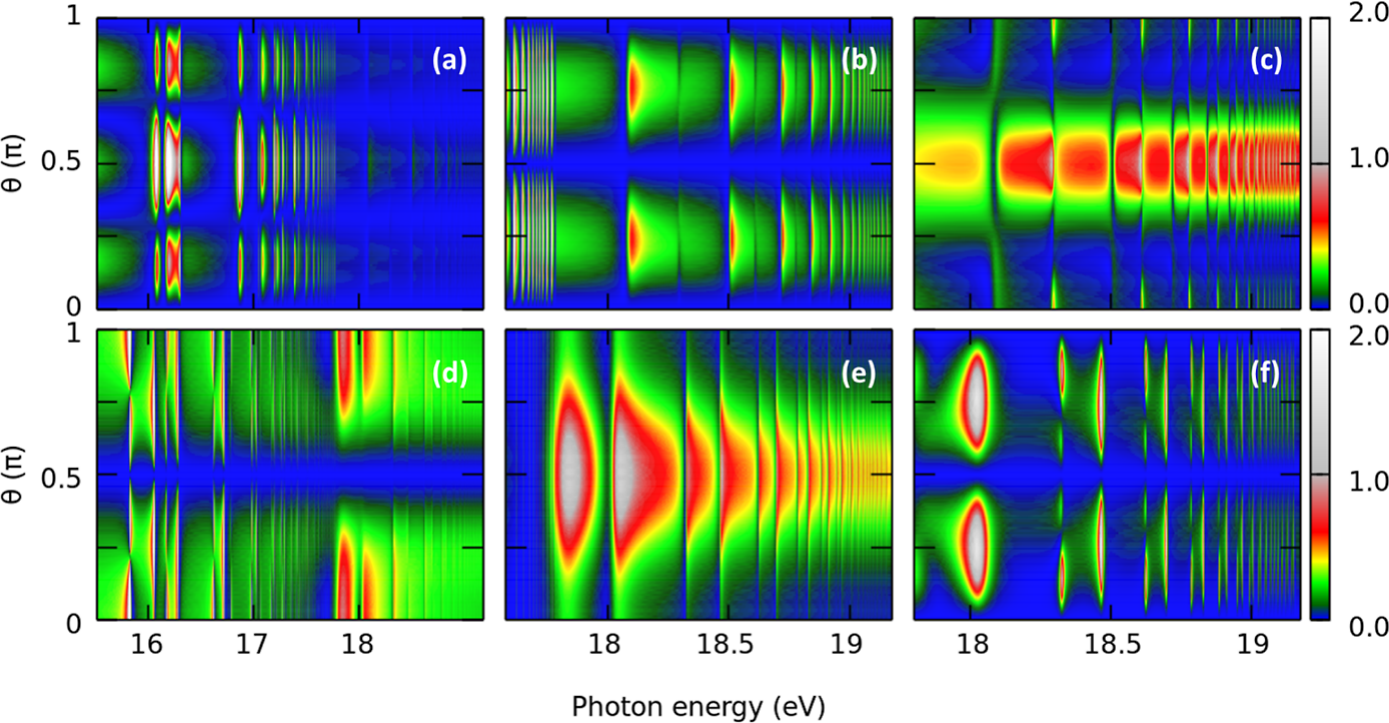
Molecular frame photoelectron angular distributions, at a fixed azimuthal angle ϕ, as a function of the photon energy. Top row: Molecular axis is placed parallel to the light polarization vector, with ϕ = 0, for the channels (a) X^2^Π_g_, (b) A^2^Π_u_, and (c) B^2^Σ_u_^+^. Bottom row: Molecular axis is placed perpendicular to the light polarization vector, with ϕ = π/2, for the channels (d) X^2^Π_g_, (e) A^2^Π_u_, and (f) B^2^Σ_u_^+^. Results have been normalized by a constant factor for a better visualization.

We have also evaluated molecular-frame photoelectron angular distribution, i.e., cross sections resolved in molecular orientation and photoelectron emission angle with respect to the polarization direction, with particular emphasis on the range of photon energies between the first and fourth ionization thresholds. As we have seen, this energy region features multiple series of autoionizing Rydberg states. The MFPAD is very sensitive to electron correlation in the vicinity of autoionizing states and thus requires a fully correlated treatment of both the target electronic states and the electronic continuum. Figure 7a–c depicts the MFPADs for photoionization from the ground state leading to states of ^1^Σ_u_^+^ symmetry, i.e., the molecular axis is placed parallel to the light polarization vector, as a function of the photon energy. Each panel presents the MFPADs at a fixed azimuthal angle ϕ = 0 associated with the X^2^Π_g_, A^2^Π_u_, and B^2^Σ_u_^+^ cation states, respectively. Figure 7d–f depicts the corresponding MFPADs at a fixed azimuthal angle ϕ = π/2 for photoionization from the ground state leading to states of ^1^Π_u_ symmetry, i.e., the molecular axis is placed perpendicular to the light polarization vector. In general, for a given ionization channel, the off-resonance MFPADs exhibit a smooth behavior as a function of photon energy. The photoelectrons are mainly ejected in a preferential direction for all photon energies. In contrast, in the vicinity of a resonance, the MFPADs experience strong variations as the photon energy crosses their energy position. This effect is a direct consequence of the sudden change in the phase of the scattering state describing the photoelectron at resonance. This change in the phase modifies the ratio between the partial cross sections. This ultimately leads to a different dominant partial wave and thus to an abrupt change in the MFPADs. This effect has been extensively studied in both atomic and molecular systems.^20,21,57,58^ As observed in Figure 7c, the off-resonance photoelectron is primarily emitted at 90°. However, the on-resonance photoelectrons are mainly ejected at 0 and 180°. Examination of the partial cross sections (not shown here) suggests that the ε*s* + ε*d* and the ε*s* + ε*g* channels dominate in the vicinity of the 4σ_g_^–1^*np*σ_u_ and 4σ_g_^–1^*nf*σ_u_ resonances, respectively, while the ε*s* channel becomes the prominent channel elsewhere. A similar analysis can be made for the different ionization channels, although the presence of overlapping resonances makes the interpretation more difficult.

**Figure 8:**
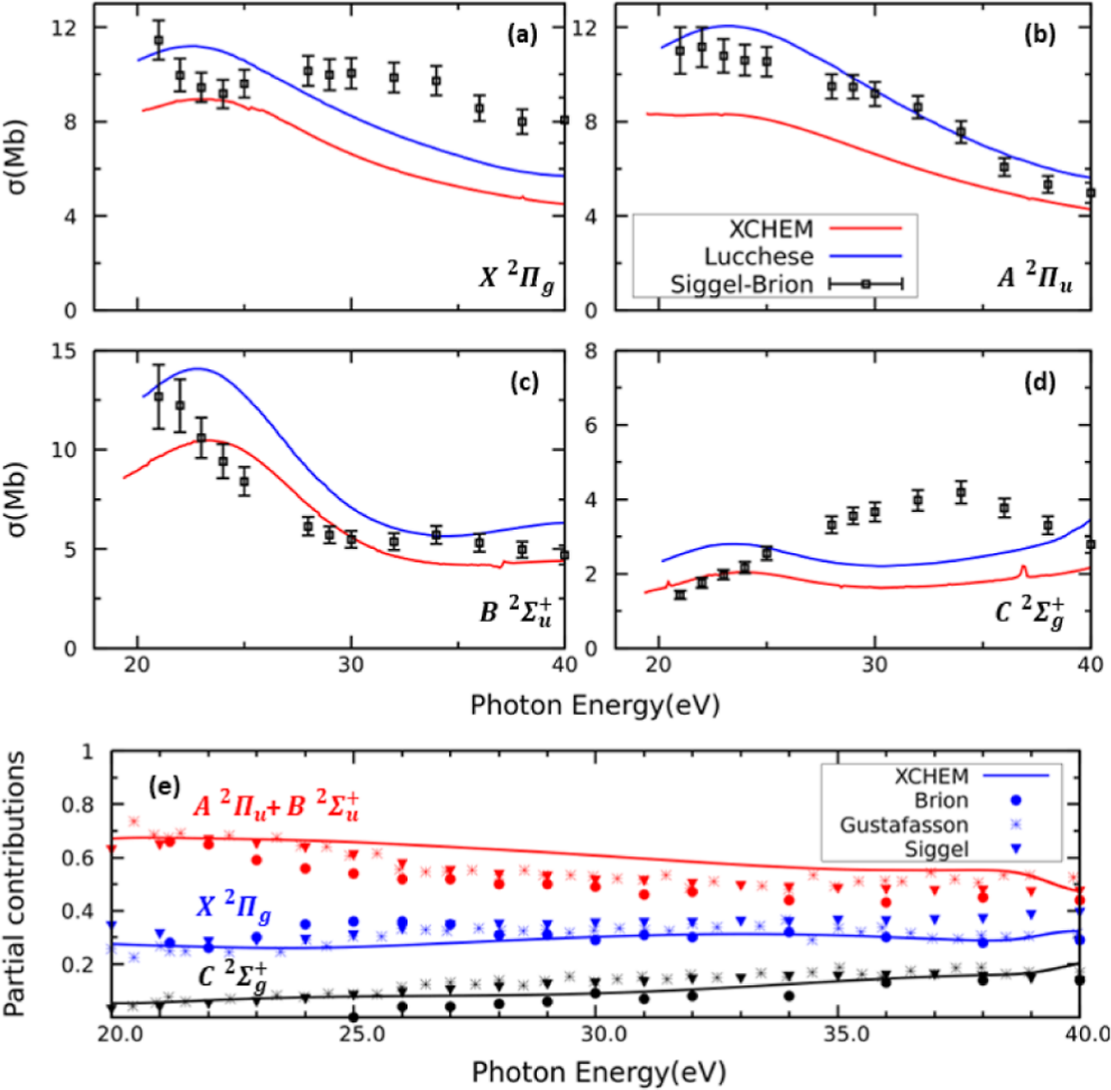
(a) Total cross section for single-photon ionization as a function of the photon energy. Panels (a)–(d) depict the cross section correlated to each ionization channel (see insets). Black points: Experimental results from refs (34) and (37). Solid blue line: Theoretical from ref (40). (e) Branching ratios for various ionization channels compared with the experimental results from refs (34), (35), and (37).

**Figure 9:**
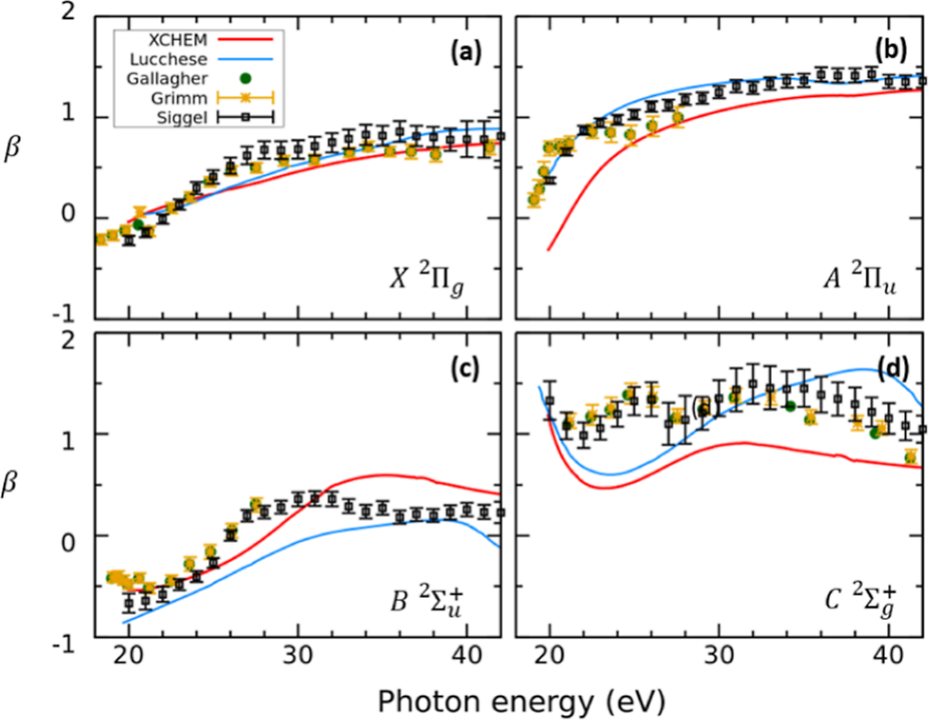
Photoelectron angular distribution asymmetry parameter β for single-photon ionization as a function of the photon energy. Each panel depicts the β parameter correlated to each ionization channel (see insets). Yellow asterisks, green dots, and black squares are experimental results from refs (36) and (38) and, ref (37), respectively. Blue dots: theoretical results from ref (40). Red line: Presents results.

Finally, for the sake of completeness, Figure 8a–d shows the total cross sections associated with leaving the cation in each of the four included channels for photon energies 20 eV ≤ ℏω ≤ 40 eV. This energy region has been extensively studied both theoretically and experimentally.^34−42^ The present results are compared with experimental data obtained using synchrotron radiation measurements^34−38^ and theoretical results calculated using Hartree–Fock static-exchange potentials.^40^ The corresponding β asymmetry parameters are presented in Figure 9a–d. The cross sections and the β asymmetry parameters are generally in good agreement with the experimental data, in particular for the X^2^Π_g_, A^2^Π_u_, and B^2^Σ_u_^+^ cation states. In contrast, the cross section for the C^2^Σ_g_^+^ cation state deviates somewhat from the experimental data. Although the present calculations do not include averaging over the vibrational motion of the molecule, previous calculations seem to indicate that the inclusion of the effects of the nuclear motion would not alter considerably the cross sections in this energy region.^39,41^ On the other hand, calculations reported in ref (42) including up to 96 channels in the close-coupling expansion exhibit a very good agreement with the experiment in this energy region. Therefore, the reason for the present discrepancy in the C^2^Σ_g_^+^ channel is the lack of higher ionization channels in the close-coupling expansion. For completeness, the cross section branching ratios associated with the X^2^Π_g_, A^2^Π_u_ + B^2^Σ_u_^+^, and C^2^Σ_g_^+^ states are presented in Figure 8e. The agreement between experiment and theory is excellent, despite the discrepancy found for the C^2^Σ_g_^+^ state cross section. As observed, photoionization in this energy region leads mainly to the X^2^Π_g_, A^2^Π_u_, and B^2^Σ_u_^+^ cation states with similar probabilities. In contrast, the population of the C^2^Σ_g_^+^ state only becomes significant for photon energies higher than 30 eV.

## Conclusions

Valence-shell single-photon ionization of the CO_2_ molecule has been theoretically studied by using the XCHEM methodology. This method makes use of a fully correlated electronic continuum and can therefore provide an accurate description of photoionization processes in the presence of Rydberg autoionizing states. We have evaluated the fully differential photoionization cross sections, with particular interest in the range of photon energies between the first and fourth ionization thresholds. This energy region features multiple series of autoionizing Rydberg states. The members of the different series have been assigned and their corresponding energy positions, autoionization widths, and quantum defects have been reported. While some of these Rydberg series have been previously observed experimentally, no theoretical description of them has been given. These results illustrate the significance of using highly correlated methods when describing photoionization processes of many-electron systems. The present calculations provide a benchmark for future attosecond pump–probe experiments in CO_2_, specifically those aiming to study the energy region between the first and fourth ionization thresholds.
